# Evolution and genomic basis of the plant-penetrating ovipositor: a key morphological trait in herbivorous Drosophilidae

**DOI:** 10.1098/rspb.2022.1938

**Published:** 2022-11-09

**Authors:** Julianne N. Peláez, Andrew D. Gloss, Julianne F. Ray, Samridhi Chaturvedi, Diler Haji, Joseph L. M. Charboneau, Kirsten I. Verster, Noah K. Whiteman

**Affiliations:** ^1^ Department of Integrative Biology, University of California, Berkeley, 94720 CA, USA; ^2^ Department of Molecular and Cell Biology, University of California, Berkeley, 94720 CA, USA; ^3^ Department of Biology and Center for Genomics and Systems Biology, New York University, New York, NY 10012, USA; ^4^ Department of Ecology and Evolutionary Biology, University of Arizona, Tucson, AZ 85721, USA; ^5^ Department of Molecular and Cellular Biology, University of Arizona, Tucson, AZ 85721, USA

**Keywords:** *Drosophila*, *Scaptomyza*, quantitative genetics, adaptation, herbivory

## Abstract

Herbivorous insects are extraordinarily diverse, yet are found in only one-third of insect orders. This skew may result from barriers to plant colonization, coupled with phylogenetic constraint on plant-colonizing adaptations. The plant-penetrating ovipositor, however, is one trait that surmounts host plant physical defences and may be evolutionarily labile. Ovipositors densely lined with hard bristles have evolved repeatedly in herbivorous lineages, including within the Drosophilidae. However, the evolution and genetic basis of this innovation has not been well studied. Here, we focused on the evolution of this trait in *Scaptomyza*, a genus sister to Hawaiian *Drosophila*, that contains a herbivorous clade. Our phylogenetic approach revealed that ovipositor bristle number increased as herbivory evolved in the *Scaptomyza* lineage. Through a genome-wide association study, we then dissected the genomic architecture of variation in ovipositor bristle number within *S. flava*. Top-associated variants were enriched for transcriptional repressors, and the strongest associations included genes contributing to peripheral nervous system development. Individual genotyping supported the association at a variant upstream of *Gαi*, a neural development gene, contributing to a gain of 0.58 bristles/major allele. These results suggest that regulatory variation involving conserved developmental genes contributes to this key morphological trait involved in plant colonization.

## 1. Introduction

Herbivorous insects are among the most successful animal radiations, representing approximately one-quarter of animal species, yet are only found in one-third of extant insect orders [1–3], suggesting phylogenetic constraint on adaptations required for this transition. Indeed, herbivory requires multi-faceted adaptations, including locating appropriate host plants, attachment to the host, resisting desiccation, and feeding on nutritionally unbalanced, chemically and physically defended plant tissues [4]. Despite the paucity of insect orders with herbivorous species, herbivory has evolved many times independently within some orders [2], including at least 25 times within Diptera [5]. Identifying whether these clades share specific traits may help resolve the paradox of why herbivory has only evolved in some orders despite often leading to species radiations.

The plant-penetrating ovipositor is one such trait that facilitates entry into this new ecological niche and has driven species radiations. It evolved within major radiations of true fruit flies (Tephritidae), leaf-mining flies (Agromyzidae) and leafhoppers (Cicadellidae)—together comprising approximately 27 500 species—as well as within sawflies (Tenthredinidae), katydids (Tettigoniidae) and plant bugs (Miridae) [6]. The insertion of eggs into plant tissue allows neonate larvae to bypass physical defences and hatch directly into the leaf interior, avoiding dessication and providing protection from the environment and enemies [4,7]. Some insects with plant-penetrating ovipositors, like agromyzid flies, also consume leaf exudates from oviposition wounds [8], providing a novel trophic resource to adults, even in the absence of chewing mouthparts.

**Figure 1.**
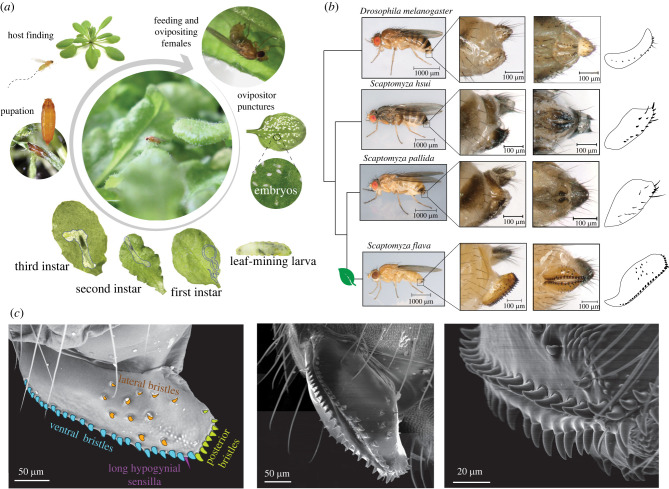
Female ovipositor morphology of the herbivorous drosophilid *Scaptomyza flava* enables cutting into tough plant tissues for feeding and egg-laying*.* (*a*) The *S. flava* life cycle is strongly dependent on accessing the leaf interior. On the underside of an *Arabidopsis thaliana* leaf, a female uses her serrated ovipositor to create a leaf puncture to drink from and/or to oviposit in. Larval mines outlined in blue. (*b*) The comparison of ovipositors (insets) of herbivorous and non-herbivorous drosophilid species. (c) Scanning electron micrographs of the ovipositor of *S. flava* (hypogynial short sensilla, not shown). (Online version in colour.)

The Drosophilidae is a compelling species radiation for studying plant-penetrating ovipositors as a key morphological trait for the evolution of herbivory. While most drosophilid species feed on decaying plant tissues and microbes, plant-penetrating ovipositors are found in all known lineages that evolved herbivory independently: (i) *D. suzukii*, a generalist pest of ripe fruit [9], (ii) leaf-miners within the genus *Scaptomyza* (Drosophilidae)*,* which includes the model herbivore *Scaptomyza flava,* a specialized pest of Brassicaceae crops (figure 1*a*) [10], (iii) *Scaptodrosophila notha*, a specialist of living bracken fern fronds (*Pteridium* spp.) [11] and (iv) leaf-mining species of *Lordiphosa* [12]. All four lineages bear sclerotized ovipositors, studded with sharp, enlarged bristles used to pierce or scrape into living plants. Drosophilid flies have already been in use as models for the evolution of herbivory [13], and genetic dissection of herbivore-specific traits is enabled by the availability of high-quality genome assemblies across the genus [14], functional genetic data from *Drosophila melanogaster*, and a strong phylogenetic framework for Drosophilidae [15].

Although ovipositors of herbivorous drosophilids differ in many morphological aspects, one shared feature is a row of supernumerary bristles along the ventral and posterior margins used for cutting (e.g. Figure 1*b*,*c*). Drosophilids possess several ovipositor sensilla types, including one long hypogynial sensilla and three trichoid short hypogynial sensilla located on the inner surface of the oviscapt apex [16]. On the outer surface are hair- or peg-like ovipositor bristles (also termed hypogynial teeth), located ventrally, posteriorly and/or laterally (figure 1*c*). While the first two sensilla types on the inner surface do not vary widely in number across species, ovipositor bristles vary significantly in number, shape and location [17]. The function of these bristles in *Drosophila* is not fully understood, but in *D. suzukii* and *D. melanogaster*, ovipositor bristles harbour mechanosensory neurons, and are used to sense substrate hardness during egg-laying [18,19]. We thus hypothesize that during the evolution of herbivory, the ovipositor was co-opted into a cutting tool through the increased number and hardening of these mechanosensory bristles, facilitating access to turgid, fibrous plant cells. We therefore focused on increased bristle number, which has been well studied from a quantitative genetics and developmental biology perspective [20].

To investigate whether increased ovipositor bristles were associated with this transition to herbivory and to explore underlying molecular mechanisms of this increase, we focused on the cutting ovipositor of herbivorous *Scaptomyza,* particularly *S. flava.* In addition to morphological changes, *S. flava* has acquired a stereotyped behavioural repertoire for feeding and egg-laying: females tap their ovipositors around the leaf searching for an ideal site, then scoop a hole by repeatedly opening their oviscapts laterally, and finally turn anticlockwise to imbibe the leaf exudates (electronic supplementary material, videos S1 and S2). Because *S. flava* females create hundreds of punctures over their lifespan [21] and spend a lengthy amount of time cutting each hole—roughly one minute versus a few seconds in microbe-feeders, such as *D. melanogaster* [22]—adaptations that reduce energy expenditure could be favoured by natural selection. Additionally, plants vary in the toughness of their leaves, which may correlate with variation in ovipositor bristle number in an optimal foraging context. Neonate *S. flava* larvae are also dependent on oviposition directly into the leaf, as those that hatch outside do not survive [10].

We first investigated whether ancestral increases in ovipositor bristle number paralleled the transition to herbivory in *Scaptomyza*, using phylogenetic generalized least-squares (PGLS) methods and ancestral state reconstruction (ASR). Then, to uncover candidate genes and developmental pathways that underlie variation in ovipositor bristle number, we used pooled genome-wide association mapping (pool-GWAS) [23,24] within the herbivorous species *S. flava*. Finally, we sought to confirm our pool-GWAS results by genotyping individuals and estimating the effect size of a single-nucleotide polymorphism (SNP) that reached genome-wide significance.

## 2. Materials and methods

### (a) Phylogeny reconstruction

We estimated a phylogeny of *Scaptomyza,* including the sister clade of Hawaiian *Drosophila*, using 11 genes and 95 taxa (electronic supplementary material, table S1). We expanded a previous phylogenetic dataset [25] with five additional taxa: two with sequenced genetic markers (*S.* nr. *nigrita* (Nevada) and *S. montana* (Arizona) [26]), and three obtained in this study from California (*S.* nr. *nigrita*, *S. montana*, and an undescribed species *S.* sp). DNA extraction and PCR methods have been described previously [27]. PCR amplicons were cleaned and Sanger sequenced in both directions, and trimmed and manually aligned to the other taxa using MAFFT v7.450. We estimated a species tree using the alignment of concatenated genes by maximum likelihood (ML) in RAxML [28], and a time-calibrated tree by Bayesian inference using MrBayes v.3.2.4 [29] and BEAST v.2.4.6 [30]. Alignment partitioning and model implementation are described in the electronic supplementary material, Methods. Complete phylogenies are reported in the electronic supplementary material, figures S1 and S2.

### (b) Ovipositor trait evolution

To test whether ovipositor bristle number changed significantly during the evolution of herbivory, we performed PGLS regression [31], including the following predictor variables: larval diet (herbivorous versus non-herbivorous) [27], ovipositor length, phylogenetic relatedness and source of bristle counts (literature versus this study). We collected bristle counts from illustrations or images from the literature, or directly from wild or laboratory-reared individuals (electronic supplementary material, table S2). Where available, we averaged across multiple individuals and literature sources. Because distinguishing between sensilla types from the literature was not always clear, we counted all visible bristles and hypogynial long sensilla on one oviscapt, omitting the inconspicuous hypogynial short sensilla. We obtained ovipositor lengths from literature sources either from published measurements, or using provided scale bars. Ovipositors of wild and laboratory-reared flies (*n* = 2–10 per species) were mounted on slides with Permount mounting medium (Fisher Scientific) and coverslips, and photographed using an EOS Rebel T3i camera (Canon) mounted onto a Stemi 508 stereo microscope (Zeiss) with a 1000 µm scale bar. Ovipositor length was then measured using ImageJ.

We performed PGLS regression using ape [32] and picante [33] packages in R. Comparing models of trait evolution (Brownian motion, Ornstein–Uhlenbeck, early burst, and white noise) for bristle number using AICc in the geiger R package [34], we selected Brownian motion as the best fit (electronic supplementary material, table S3). The degree of phylogenetic signal in the residuals was estimated using Pagel's lambda (*λ*) [35]. To visualize correlated evolutionary changes in diet and bristle number, we mapped onto the phylogeny estimated ancestral states of both traits by ML using phytools [36] and ape [32]. We compared models of trait evolution (equal rates, symmetric and all rates different) for larval diet and identified equal rates as the best fit (electronic supplementary material, table S4).

### (c) Mapping population and measurements for pooled genome-wide association mapping

To identify genetic polymorphisms contributing to variation in bristle number, we used a pool-GWAS to detect allele frequency differences between pools of individuals with extreme phenotypes from the same population. Two *S. flava* outbred laboratory populations were founded from collections on mustard plants, one larvae per plant, in Portsmouth, NH, USA (both within 0.1 km of 43.10068, −70.81246): one population (NH1) was founded from 79 larvae from *Turritis glabra* and the second (NH2) from 58 larvae from *T. glabra* and *Barbarea vulgaris*. Within each population, newly eclosed adults were transferred to one mesh cage containing *Arabidopsis thaliana* (Col-0 accession) and allowed to mate randomly. In each population, over 1200 offspring (G1) were reared on a mixture of *T. glabra* and *B. vulgaris*, allowed to mate randomly, and adult female offspring (G2) were preserved in 95% ethanol and phenotyped for the GWAS (electronic supplementary material, figure S3 illustrates the mating scheme). (Flies were collected and bred on different hosts for a separate study on host adaptation.)

We mounted ovipositors on slides as described above, counting only ventral bristles (figures 1*c* and 3*a*), summed across both oviscapts. We excluded posterior bristles, which were largely invariable in number, and lateral bristles because we speculated that their involvement in leaf-cutting may be limited due to their smaller size. We quantified ovipositor length as described above, and also wing chord (proxy for body size), measured from the wing base to the apex following the third longitudinal vein (figure 3*a*). Two independent measurements were averaged per specimen. Linear regression analyses in a pilot experiment (NH1/NH2 flies, *N* = 100) revealed that bristle number was positively correlated with ovipositor length (*B* = 0.097 [s.e. = 0.025] pegs per micrometre length, *R*^2^ = 0.134, *p* = 0.0001), but not wing length (*B* *=* 0.001 [s.e. = 0.002], *p* = 0.25). We therefore quantified both ovipositor length and bristle number for all individuals (NH1, *N* = 308 flies; NH2, *N* = 422 flies).

Narrow-sense heritabilities of ovipositor length and bristle number were quantified using mother–daughter regression; details are presented in the electronic supplementary material, Methods.

### (d) Pooled genome sequencing

Flies in the NH1 and NH2 populations were split into two phenotypically extreme pools per population (four pools: NH1-low, NH2-low, NH1-high, NH2-high), composed of 60–85 females in the upper or lower 20% tail of the distribution of residual bristle number. Residual bristle number was determined through a linear regression of ovipositor bristle number against ovipositor length using the *lm* function in R. Flies were homogenized with stainless-steel beads and a TissueLyser (Qiagen). Genomic DNA was extracted using a DNeasy Blood and Tissue Kit (Qiagen). One Illumina library per pool was constructed with 100 bp paired-end reads and a 350 bp insert size. Each library was sequenced on one half lane on an Illumina HiSeq 2500 at Arizona State University.

### (e) Read mapping, pooled genome-wide association mapping and gene ontology enrichment analysis

Illumina reads were mapped to the *S. flava* reference genome (GenBank accession no. GCA_003952975.1) and filtered following best practices for pooled genome sequencing [37]. Statistical significance of between-pool allele frequency differences per site was estimated using the Cochran–Mantel–Haenszel test [38]. We identified and conservatively sought to correct for a slight inflation of *p*-values [39]. However, because the *p*-value distribution was non-uniform with an excess of higher and lower values, typical corrections based on the observed versus median test statistic gave unsuitable inflation factors. We therefore regressed observed against expected –log_10_(*p*) values with the intercept constrained to 0 and divided each –log_10_(*p*) value by the slope of the regression line [40]. Further details are presented in the electronic supplementary material, Methods.

To identify genes located in or near the top SNPs (ranked by *p-*value), we located the nearest annotated gene in either direction, using genome-wide annotations for *S. flava* [10]. We checked for unannotated genes between the SNP and closest annotated gene by comparing the spanning sequence against the *D. melanogaster* RefSeq protein database, using NCBI BLASTx with default settings. Gene functions were gathered from the Gene Summary, Gene Ontology Annotations and linked publications in Flybase (release 2020_01) [41]. To better interpret the pool-GWAS results, we profiled linkage disequilibrium (LD) and population structure in several wild populations of *S. flava* in Massachusetts and New Hampshire. Further details are presented in the electronic supplementary material, Methods.

To determine if any predicted functions were overrepresented among genes intersecting the top GWAS associations, we performed a Gene Ontology enrichment test using GOWINDA, which implements a permutation-based approach tailored to the properties of GWAS datasets [42]. Full details, including orthology-based functional annotation and extension of gene models to capture regulatory regions, are described in the electronic supplementary material, Methods.

### (f) Reproducing pooled genome-wide association mapping association for a candidate single-nucleotide polymorphism

Pool-GWAS can be confounded by uneven contributions of individuals to pools and biases in sequencing and read mapping. To verify our pool-GWAS results using an approach robust to these confounding factors, we genotyped individual females at one of the top SNPs and estimated its effect size. The SNP was chosen because of its close proximity to *G alpha i subunit* (*Gαi*), a gene involved in asymmetric cell division of sensory organ precursor (SOP) cells from which bristles are derived [43]. Ovipositor bristle number and length were measured as described above. Genomic DNA was extracted from 74 females (NH1/NH2 (G2), electronic supplementary material, table S5), and a target region of 500 bp around the SNP was Sanger sequenced. Bristle number was modelled in a generalized linear model, assuming an additive effect of the major allele, using the *lm* function in R. Additional details are presented in the electronic supplementary material, Methods.

## 3. Results

### (a) The evolution of herbivory coincided with an increase in ovipositor sensilla number within the *Scaptomyza* lineage

**Figure 2.**
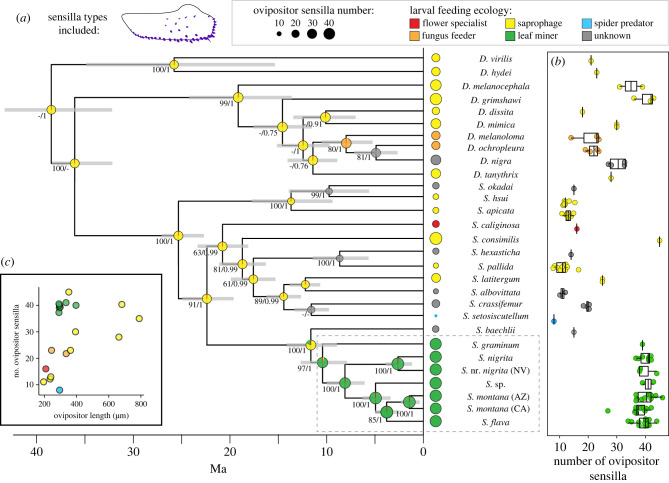
The evolution of herbivory within *Scaptomyza* coincides with an increase in ovipositor sensilla number. (*a*) Time-calibrated phylogeny of herbivorous *Scaptomyza* and their non-herbivorous relatives, based on ML and Bayesian analyses, using 95 taxa, 11 genes and fossil and biogeographic time calibrations. Only taxa with data on ovipositor sensilla number and diet are shown (*N* = 29; see electronic supplementary material, figures S1 and S2 for full phylogeny). Branch support is indicated by ML bootstrap values (greater than or equal to 50%) and Bayesian posterior probability (greater than 0.9). At nodes, bars indicate 95% highest posterior density interval around mean node age, pie graphs show probabilities of ancestral larval diets and circle size represents ancestral ovipositor sensilla number per oviscapt estimated from ML ASR. Average sensilla numbers for extant species are shown at the tips, with individual counts shown in (*b*). (*c*) Scatterplot of ovipositor sensilla number as a function of ovipositor length. (Online version in colour.)

PGLS methods revealed that ovipositor sensilla number was strongly influenced by larval diet (herbivorous versus non-herbivorous) (*F*_1,19_ = 5.801, *p* = 0.028), phylogenetic relatedness (Pagel's *λ* = 1) and by ovipositor length (*F*_1,19_ = 4.655, *p* = 0.047), but not by source type (literature versus this study) (*F*_1,19_ = 1.401, *p* = 0.254) (electronic supplementary material, table S6). ASRs of ovipositor sensilla number and larval diet similarly suggested that ovipositor sensilla number increased coincident with the evolution of herbivory in *Scaptomyza*, estimated approximately 10.4 million years ago (Ma) (8.2–13 Ma, 95% highest probability density) (figure 2*a*; electronic supplementary material, figure S4). Relative to interspecific differences, variation within species was low (figure 2*b*).

### (b) Genome-wide association mapping on ventral ovipositor bristle number in *Scaptomyza flava*

**Figure 3.**
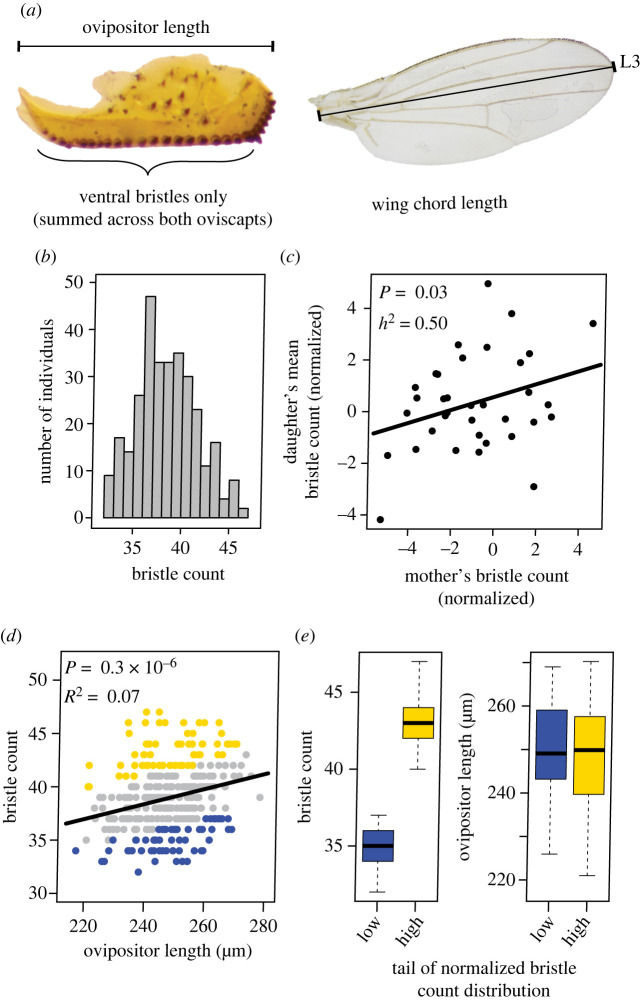
Ovipositor bristle number is continuously distributed and heritable in *S. flava*, enabling quantitative genetic dissection*.* (*a*) Ovipositor bristle counts for this analysis included only ventral bristles. Wing chord length was measured along the third longitudinal vein (L3). (*b*,*d*,*e*) Phenotype distributions of the pool-GWAS outbred mapping population (NH1). (*b*) Ovipositor bristle number follows a continuous distribution*.* (*c*) Ovipositor bristle count, expressed as residuals from a linear regression of bristle count against ovipositor length, is heritable in the narrow sense (*h*^2^ = 0.50) from mother–daughter regression analysis (*N* = 35). (*d*) After regressing out the effect of ovipositor length on bristle count, pools of phenotypically extreme individuals included either the upper (yellow) or lower (blue) 20% distribution tails. (*e*) Low pool individuals had approximately 20% fewer bristles than those in the high pool, but not statistically different ovipositor lengths. (Online version in colour.)

Variation in ovipositor bristle number followed a continuous distribution in the NH1 and NH2 outbred laboratory populations of *S. flava* (figure 3*a,b*), typical of a quantitative trait controlled by multiple loci. Linear regression of ovipositor bristle number from mother–daughter pairs, controlling for the effect of ovipositor length, revealed that additive genetic variation accounted for half of this phenotypic variation (*p* = 0.034, *h*^2^ = 0.50 ± 0.27 s.e.; figure 3*c*). By contrast, variation in ovipositor length was not heritable (*p* = 0.31).

**Figure 4.**
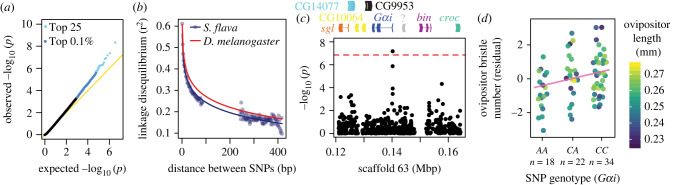
Pool-GWAS for variation in *S. flava* ovipositor bristle number implicates genes involved in nervous system development. (*a*) An excess of strong *p*-values (conservatively adjusted for genomic inflation) suggests an enrichment of true associations among the top-scoring SNPs. (*b*) The relationship between physical distance and LD, inferred from pooled sequencing of wild *S. flava* from Belmont, MA, USA, is similar to that seen in *D. melanogaster*. The distribution gap reflects the difference in read insert size (350 bp) and sequenced read length (100 bp). (*c*) Manhattan plot centred on a top SNP upstream of *G-alpha i subunit* (*Gαi*), a gene involved in neural development [43]. The red line indicates the 5% FDR cutoff for genome-wide significance. Annotated genes are plotted above. (*d*) Genotyping individuals for the SNP near *Gαi* recapitulate the pool-GWAS association. Bristle number, expressed as residuals (observed–predicted based on covariates), increased additively and independently of ovipositor length, shown by colour scale. Regression line is shown in pink. (Online version in colour.)

We next sought to characterize the genomic architecture underlying this variation using a pool-GWAS. Because ovipositor length was positively correlated with bristle number (figure 3*d*), pools were constructed by adjusting bristle number relative to that expected from ovipositor length (figure 3*e*). This approach should interrogate bristle number independently of ovipositor size and minimize noise from non-heritable variation in ovipositor length. Mapped reads from whole genome re-sequencing of the four pools had a mean experiment-wide coverage depth of 166X per polymorphic site (per pool: NH1-low: 31x; NH1-high: 38x; NH2-low: 23x; NH2-high: 53x). After excluding low-frequency variants (1.6 million SNPs remaining), we found an excess of SNPs with significantly differentiated allele frequencies among high- and low-bristle number pools (figure 4*a*), with five and 19 significant SNPs at 5% and 10% false discovery rate (FDR) cutoffs, respectively (table 1; electronic supplementary material, table S7). Because LD decays in *S. flava* at a rapid rate like that seen in *D. melanogaster* (figure 4*b*), SNPs showing strong associations are likely in close proximity to causal polymorphisms or are causal themselves. LD decay rates were similar across several populations in Belmont, MA and Portsmouth, NH, and across two host species (*T. glabra* and *B. vulgaris*) (electronic supplementary material, figure S5). We cannot fully discount the possibility that long-range LD caused by undetected population structure could cause false associations between ovipositor bristle number and candidate SNPs. However, we did not find evidence for genetic population structure (*F*_st_) across these populations (electronic supplementary material, figure S6).

**Table 1.** Top SNPs associated with variation in ovipositor bristle number are located in or near genes involved in the development of bristles, cuticles and the nervous system*.* SNPs reaching genome-wide significance (FDR ≤ 0.05) from the pool-GWAS are shown in descending *p*-value ranking.

*p*-value ranking	Scaffold [position]	*p*-value	FDR *q* value	nearby gene(s)	SNP location (relative to gene)		gene product function (from *D. melanogaster*)
1	465 [184]	4.66 × 10^−09^	0.008	muscle-specific protein	453 bp	downstream	actin binding; cytoskeleton organization; required for proper positioning of muscle nuclei, mitochondria, and neuromuscular junction
2	15 [186,025]	4.26 × 10^−08^	0.036	heavyweight	8623 bp	downstream	predicted to have phosphotyrosine residue binding activity; polymorphisms associated with body mass and starvation resistance
				cuticular protein 11B	1625 bp	downstream	chitin-based cuticle development
3	63 [140,206]	6.69 × 10^−08^	0.037	G protein alpha i subunit	2 bp	upstream	asymmetric neuroblast division and asymmetric protein localization involved in cell fate determination; cytoskeleton organization; and nervous system development
				Don juan-like [*D. grimshawi*]	3446 bp	downstream	unknown
4	53 [135,098]	1.20 × 10^−07^	0.048	sloppy paired 2	560 bp	upstream	transcription factor that regulates embryonic segment polarity and neural fate specification by temporal patterning of medulla neuroblasts
				CG11018	1809 bp	upstream	unknown
5	71 [290,082]	1.42 × 10^−07^	0.048	CG32655	9932 bp	downstream	unknown
				tenascin accessory	42 315 bp	downstream	nervous system development; regulation of cell-cell adhesion; and synapse organization

Inferences about gene function were based on orthologous function in *D. melanogaster* from Flybase FB2020_01.

Many of the top SNPs (electronic supplementary material, table S7), including those reaching genome-wide significance (FDR ≤ 0.05, table 1), were located near genes involved in neural development or neural cell fate specification (i.e. *G protein alpha i subunit, sloppy paired 2, tenascin accessory*), cytoskeleton organization (i.e. *muscle-specific protein*) and cuticle development (i.e. *cuticular protein 11B*).

### (c) Gene ontology enrichment analysis on candidate single-nucleotide polymorphisms

To gain insight into developmental mechanisms that may contribute to variation in ovipositor bristle number, we tested for enriched gene ontology (GO) annotations among genes intersecting SNPs with the strongest GWAS associations (top 0.1% and 0.005% of *p*-values genome-wide). Using a restricted set of GO terms to minimize redundancy, we uncovered a single enriched term: RNA polymerase II-specific DNA-binding transcription repressor activity (GO:0001227; table 2; electronic supplementary material, table S8). Many of the transcriptional repressors identified fine-tune gene expression levels during the specification of cell fate during neural development. Notably, the strongest GWAS association among transcriptional repressors fell within the gene *hairy* (*h*), which in *D. melanogaster* functions in the establishment of bristle precursor positioning from within proneural clusters [44].

**Table 2.** GO terms enriched among genes intersecting the most significant pool-GWAS SNPs.

candidate SNPs	GO terms investigated		genes intersecting candidate SNPs					*p* (bonf.)	genes intersecting candidate SNPs
		enriched GO term	obs.	exp.	possible^a^	fold enrichment	*P*		
top 0.1%	non-redundant subset	DNA-binding transcription repressor activity, RNA polymerase II-specific (Molecular Function, level 4; GO:0001227)	16	5.469	63	2.93	0.00004	0.0092	*aop, chn, dpn, E(spl)mβ-HLH, E5, h, Hey, HHEX, l(2)gd1, lms, Mad, Med, Rbf2, CG12299, CG1233, CG7987*
top 0.1%	full set	phosphatidylinositol biosynthetic process (Biological Process, level 6; GO:0006661)	8	2.013	43	3.97	0.00067	1.00	*GAA1, Pi3K68D, PIG-O, PIG-S, PIG-Z, PIP5K59B (x2), CG5342*
top 0.005%	full set	establishment of cell polarity (Biological Process, level 3; GO:0030010)	5	0.751	92	6.66	0.00075	1.00	*Gαi, Khc-73, scrib, sktl, CG5964*
Gene functions of SNPs enriched in DNA-binding transcription repressor activity, RNA polymerase II-specific (GO:0001227), the only significantly enriched GO term from the analysis using non-redundant terms									
Scaffold	genes intersecting SNPs	-log10(*p*)	genome- wide rank^b^	SNP location	transcriptional repressor function (from *D. melanogaster*)			nervous system dev.	Notch signalling pathway
363 [49,064]	*aop*	3.417	1531	120 bp upstream	regulates cell fate transitions during development of the nervous system, heart, trachea and eye			✓	
96 [260,245]	*chn*	4.190	379	1255 bp upstream	regulates *emc, h, ac* and *Dl*. Functions in sensory neurons, photoreceptors, blood cells, and muscle and intestinal precursors			✓	
160 [26,969]	*dpn*	4.023	509	within 3rd exon	regulates genes requiring bHLH protein for transcription. Maintains self-renewal and identity of type II neuroblasts in larval brain			✓	✓
196 [39,864]	*E(spl)mβ-HLH*	4.217	369	973 bp downstream	regulates genes requiring bHLH protein for transcription, including *achaete–scute* complex (AS-C) genes. Contributes to neural-epidermal lineage decision during neurogenesis			✓	✓
521 [31,530]	*E5*	4.429	237	within 1st intron	predicted to be involved in brain development and neuron differentiation			✓	
328 [130,182]	*h*	5.763	23	within 1st exon	bHLH transcriptional repressor that recruits Gro corepressor to target promoters. Contributes to embryonic segmentation and peripheral neurogenesis			✓	
12 [430,878]	*Hey*	3.521	1269	within 2nd intron	*Hairy/E(spl)-related with YRPW motif* (*Hey*) encodes a bHLH transcription factor involved in neuron fate determination			✓	✓
115 [115,881]	*HHEX*	4.157	405	within 1st intron	involved in response to sucrose. One allele shown to affect trichogen cells, which generates the sensillum shaft			✓	
216 [115,550]	*l(2)gd1*	3.420	1520	within 3rd exon	involved in female germ-line stem cell asymmetric division; wing vein morphogenesis; and sensory organ precursor cell division			✓	✓
332 [46,880]	*lms*	4.252	351	1107 bp downstream	homeodomain transcription factor				
751 [23,120]	*Mad*	4.381	266	within 1st intron	mediates response to BMP ligands encoded by *dpp, scw* and *gbb* (proteins involved in growth regulation, patterning and stem cell fate)			✓	
302 [38,793]	*Med*	3.696	943	2496 bp downstream	binds to Mad or Smox to facilitate signal transduction for *dpp* or Activin ligands. Involved in dorsal–ventral patterning, patterning/proliferation of wing disc and gene expression in larval brain mushroom body			✓	
1 [1,171,740]	*Rbf2*	3.418	1528	184 bp upstream	cell cycle and developmental regulator. Represses the expression of differentiation markers in ovaries and embryos				
394 [52,743]	*CG12299*	3.442	1466	901 bp downstream	predicted to enable DNA-binding transcription repressor activity				
355 [77,106]	*CG1233*	4.414	244	within 3rd exon	predicted to enable DNA-binding transcription repressor activity				
222 [139,175]	*CG7987*	5.364	39	957 bp downstream	predicted to enable DNA-binding transcription repressor activity				

^a^The maximum possible number of intersections equals the number of genes assigned to the GO category that have at least one genotyped SNP passing quality control filters.

We further tested for enrichment using the exhaustive list of all GO terms. Although no terms were enriched after applying a strict Bonferroni correction, two terms surpassed a nominal cutoff of *p* < 0.001, and both reflect broadly conserved developmental functions: phosphatidylinositol (PI) biosynthetic process and establishment of cell polarity (table 2; electronic supplementary material, table S8). Many of the candidate genes annotated with PI biosynthetic process (GO:0006661) are kinases and transferases involved in the production of PI derivatives, which act as signalling molecules that regulate cellular growth and patterning [45,46]. Notably, the establishment of cell polarity (GO:0030010) precedes the differentiation of sensory organ precursors into distinct neural cell types through asymmetric cell division [47]. *G protein α i subunit* (*Gαi*) was one of the cell polarity genes identified and was also one of the strongest pool-GWAS associations (figure 4*c*).

### (d) Reproducing pooled genome-wide association mapping association at a top candidate single-nucleotide polymorphism (near the gene *Gαi*)

To confirm that the pool-GWAS adequately estimated allele frequencies, we focused on a SNP in the 5′ UTR of *Gαi*, one of the strongest pool-GWAS associations. We phenotyped and genotyped individual adult female flies at this locus and found that bristle number increased by 0.58 per major allele carried (*β* = 0.11 s.d., *t*_68_ = 2.88, *p* < 0.005; figure 4*d*; electronic supplementary material, table S9). This SNP explained 9.5% of the total variance in bristle number (partial adjusted *r*^2^). As expected given our study design, the SNP did not have an effect on ovipositor length (*β* = 0.02 s.d., *t*_69_ = 0.177, *p* > 0.05; electronic supplementary material, table S10). Out of five variant sites (greater than or equal to 0.05 minimum frequency) in the sequenced region, two were in strong LD with the focal SNP (electronic supplementary material, table S11). Further study will be necessary to identify the causal variant(s) in this region.

## 4. Discussion

The plant-penetrating ovipositor of herbivorous insects presents an excellent opportunity to study the evolution and genomic architecture of a complex morphological innovation, given its clear role in egg-laying and its amenability to be decomposed into simpler quantitative traits, such as ovipositor size and bristle number. We focused on ovipositor evolution in the genus *Scaptomyza,* in which herbivory has evolved relatively recently, *ca* 10.4 Ma. The wealth of data from the *Drosophila* literature made our analyses possible: genitalic morphological data available from numerous taxa to investigate evolutionary shifts in bristle number across species, and genetic and development knowledge of bristle number in *D. melanogaster* to understand the genetic architecture underlying variation at the population level in *S. flava*.

From a macroevolutionary perspective, we found that ovipositor sensilla number underwent a marked increase that coincided with the evolution of herbivory within *Scaptomyza*, a significantly larger shift than expected from the distribution of background rates of evolution across the phylogeny (figure 2*a*). Surprisingly, we also found that ovipositor sensilla number is evolutionarily malleable, repeatedly increasing and decreasing across the phylogeny, with a fivefold range across *Scaptomyza*. High variability was similarly seen within species, including a 1.5-fold range in *S. flava*. The lack of strong evolutionary constraint over both macro- and microevolutionary timescales, along with heritable standing genetic variation within populations, suggests that ovipositor sensilla number may be highly accessible to adaptive evolution. However, considering there was only one occurrence of the evolution of herbivory among sampled species, it will be necessary to test whether the same patterns exist in other independently evolved herbivorous lineages, such as those that include *D. suzukii, Scaptodrosophila notha* and herbivorous *Lordiphosa*. Further research on herbivorous drosophilids could also test whether heritable variation in ovipositor bristle number could be selected upon for divergent host use (i.e. plants of varying leaf or fruit skin toughness), as seen in fig wasps and pine-specialized sawflies [48,49].

Our phylogenetic analysis (PGLS) suggested that increased sensilla number may have evolved in addition to or partly as a result of ovipositor elongation. A longer ovipositor can accommodate more bristles, and increased ovipositor length has been studied in *D. suzukii* as a key trait to facilitate cutting into ripe fruit [50]. It will be necessary to examine whether these morphological traits are linked at molecular and developmental levels. Our GWAS in *S. flava* should have, nonetheless, targeted variation in ovipositor bristle number, rather than length, considering we used bristle counts adjusted by length, and only bristle number (not length) exhibited narrow-sense heritability (figure 3*c*).

Pinpointing genetic changes that gave rise to traits that evolved Ma can be difficult because genetic architectures may differ over short versus long timescales [51]. Still, GWAS can illuminate genes and gene functions that shape standing phenotypic variation and may contribute to evolution over longer timescales. Our GWAS results indicate that broadly conserved neurodevelopmental genes, such as *Gαi* and *slp2*, play a role in ovipositor bristle density (table 1). Genes encoding transcription repressor proteins were significantly enriched near the strongest GWAS associations, with the majority involved in neural development and neuron differentiation, and are regulated by or regulators of the Notch signalling pathway (GO:0001227, table 2). For instance, four genes (*h, E(spl)mβ-HLH*, *dpn* and *Hey*) repress basic helix–loop–helix (bHLH) proteins, which are important regulators of neurogenesis. These results are consistent with existing knowledge that insect sensilla are developmentally derived from neural precursor cells (SOPs). Bristle patterning begins with expression patterns of proneural genes, like the *achaete–scute* complex, that generate proneural cell clusters. Within these clusters, the selection of the SOP is determined by lateral inhibition mediated by Notch signalling, followed by SOP differentiation through asymmetric cell divisions into cells that form the shaft, socket and sheath, and mechanosensory and chemosensory neurons that innervate the sensilla [52]. We thus speculate that mutations in or near genes involved in SOP development could cause shifts in SOP patterning, spacing or density, thus producing more bristles. *E(spl)mβ-HLH* is a particularly strong candidate (*p*-value = 6.07 × 10^−5^), as it is directly regulated by Notch and represses the activity and expression of proneural Achaete and Scute proteins [53].

In other *Drosophila* species, genes involved in neural development also underlie differences in bristle number on the male genitalia and sexcombs of the forelegs [54]. From the same overrepresented GO category of transcriptional repressors, the top-scoring SNP (*p*-value = 1.73 × 10^−6^) was located in the gene *hairy* (*h*), a direct repressor of *achaete* [55]. RNAi knockdown of *hairy* in *Drosophila* has validated its involvement in male genitalic clasper size and bristle number, and association mapping has shown that it falls within a narrow genomic region underpinning divergence in clasper bristle number among sister species of *Drosophila* [56]. Its role in bristle and genital development, along with its contribution to intra- and inter-species variation in bristle number, make *hairy* a strong candidate for ovipositor bristle variation. It also presents an opportunity to investigate genetic parallelism for bristle number variation across the body, between sexes and across species.

Studies on the genetic architecture of adaptive traits have largely focused on monogenic, Mendelian traits with large effect loci and lower detection thresholds than genetically complex traits [57–59]. Ovipositor bristle number represents a tractable quantitative trait for genetic dissection because of its meristic nature, high variability, heritability and clear importance in facilitating entry into a new niche. Despite having a polygenic architecture similar to many quantitative traits—consisting of many, small effect SNPs—we still were able to detect a SNP with a moderately large effect (confirmed by individual genotyping). Our results suggest that pool-GWAS can be a viable method for pinpointing genomic regions that underlie quantitative trait variation. Candidate SNPs can then be interrogated through functional experimentation to understand how alternative alleles influence cell division, size expansion and reorganization during development [50]. Ultimately, this could illuminate how incremental changes could have created this key trait in herbivorous insects.

## Data Availability

All data files and scripts are available from the Dryad Digital Repository: https://datadryad.org/stash/share/q5fOC0W2LtFDayCHVQzu1ZqxBQCQLvR_WxLjXzznoGw [60]. Sanger sequences for estimating the *Scaptomyza* phylogeny were uploaded to GenBank (MH938262-MH938270). Available at NCBI sequence read archive are Illumina sequences for the pool-GWAS (SRR11252387-SRR11252390), and for evaluating LD (SRR15275350–SRR15275365; SRR20722523–SRR20722528). Sanger sequences for replicating the Gai SNP effect size were deposited on GenBank (MH884655–MH884734). Supplementary material is available online [61].
